# Supplementary material for: Herbivore biocontrol and manual removal successfully reduce invasive macroalgae on coral reefs
Source: PeerJ. 2018 Aug 8;6:e5332. doi: 10.7717/peerj.5332 (PMC6087420; doi:10.7717/peerj.5332)
Supplement: Supplemental Information 2 [file peerj-06-5332-s003.html]

DLNR Kāne’ohe Bay Invasive Algae Mitigation


Code 

- Show All Code
- Hide All Code

# DLNR Kāne’ohe Bay Invasive Algae Mitigation

#### *C Wall*

#### *3/22/2017*

## Experimental Design

Two experimental treatments were generated: **Treatment Reefs** where invasive algae were removed and urchins outplanted, and **Control Reefs**, where no mitigation action was taken.

- **Treatment Reefs: Reef 16 and 27**
- **Control Reefs: Reef 26 and 28**

Reefs were sampled through time at fixed transects; this is the **Repeated Measure** of the experiment. These sampling times were repeated twice a year, across **Seasons**: either **Winter (Nov-Feb)** or **Summer (May-June)**. The sampling periods span from 2011 to 2014. The Before period marks initial sampling where before mitigation action was taken.

- **Before: 2011 Winter**
- **After1: 2012 Summer**
- **After2: 2012 Winter**
- **After3: 2013 Summer**
- **After4: 2014 Summer**

## Statistical Design

*FIXED EFFECTS* - **Treatment** = 2 levels (Control and Treatment)  
- **Time** = 5 time points (1 Before and 4 After)

*TEMPORAL RANDOM EFFECTS* - **Transect** = the repeated measure (5 points of repeated measure)

*SPATIAL RANDOM EFFECTS* - **Reef** = Treatments or Controls are replicated at the Reef scale (n=2) - **Habitat** = Habitat is nested within Reef (1|Reef/Habitat). The sampling design at each Reef was stratified according to distinct habitat types (from crest inward): aggregate, pavement, mix).

## Community Data

Import the data, load packages, and combine all members of “invasive algae” into a common metric. Data file shows…

- **Factors** (Reef, Treatment, Habitat, Transects, Calendar Date, Time Point, Season)
- **Variables** as the proportion of community cover
- *Ed* = *Eucheuma denticulatum*
- *Ks* = *Kappaphycus alvarezii*
- *As* = *Acanthophora spicifera*
- *Gs* = *Gracilaria salicornia*
- *Ed\_Ks* = *Eucheuma* spp. and *Kappaphycus* sp.
- *Inv\_Ks* = *Eucheuma* spp., *Kappaphycus* sp., *Acanthophora* sp., *Gracilaria* sp.
- CCA = Crustose Coralline Algae
- Abiotic = Sand, Bare, Turf
- Coral = Reef corals
- NatAlgae = Native macroalgae

```
#clear work space
rm(list=ls())

#load packages
library("lme4")
library("effects")
library("car")
library("gplots")
library("plotrix")
library("ggplot2")
library("grid")
library("gridExtra")
library("scales")
library('MASS') 
library('lsmeans')
library('lmerTest')
library('lmtest')
library("lattice")
library("sjPlot")
```

```
knitr::opts_knit$set(root.dir = '~/Desktop/Research and Teaching/Invasive algae/R-stats/Invasive Algae')
ALLdata<-read.csv("data/InvAlgProjdata_DLNR.csv", header=T, na.string=NA)
# names(ALLdata)

##### examine data structure ######
# str(ALLdata)
ALLdata$Reef<-as.factor(ALLdata$Reef)
ALLdata$Date<-as.Date(ALLdata$Date, format="%m/%d/%y")
ALLdata$Time<-factor(ALLdata$Time, levels=c("Before", "After1", "After2", "After3", "After4"))
###################
```

## Figures

First, set aside the dataframe above, **ALLdata**. This original dataframe is in proportion, and this will be used in the analysis. Generate a new dataframe, **fig.df**, by converting the proportion data in ALLdata to percent cover. Finally, create a summary dataframes of means, standard error, and sample size for the percent cover data.

```
### NOTE DATA HERE IS ALL % COVER AND NOT PROPORTION.
### THIS IS OPTIMAL FOR GRAPHS, DATA SUMMARY, AND IN-TEXT SUMMARIES
### IN STATISTICAL MODELS, PROPORTION IS USED FOR AID IN TRANSFORMATION

# percent cover dataframe
fig.df<-ALLdata

fig.df$Abiotic<-(fig.df$Abiotic*100)
fig.df$As<-(fig.df$As*100)
fig.df$CCA<-(fig.df$CCA*100)   
fig.df$Coral<-(fig.df$Coral*100) 
fig.df$Ed<-(fig.df$Ed*100)
fig.df$Ks<-(fig.df$Ks*100)
fig.df$Gs<-(fig.df$Gs*100)
fig.df$NatAlgae<-(fig.df$NatAlgae*100)
fig.df$Ed_Ks<-(fig.df$Ed_Ks*100)
fig.df$Inv_Algae<-(fig.df$Inv_Algae*100)
  

# means and SE by Reef, Time for all variables
full.mean.summary<-aggregate(cbind(Inv_Algae, Ed, Ks, As, Gs, CCA, Abiotic, Coral, NatAlgae)~Reef+Treatment+Time, fig.df, mean); colnames(full.mean.summary) <- c("Reef", "Treatment", "Time", "InvAlgae-mean", "ED-mean", "Ks-mean", "AS-mean", "GS-mean", "CCA-mean", "Abiotic-mean", "Coral-mean", "NatAlgae-mean");full.mean.summary

# SE 
full.SE.summary<-aggregate(cbind(Inv_Algae, Ed, Ks, As, Gs, CCA, Abiotic, Coral, NatAlgae)~Reef+Treatment+Time, fig.df, std.error); colnames(full.SE.summary) <- c("Reef", "Treatment", "Time", "InvAlgae-SE", "ED-SE", "KS-SE", "AS-SE", "GS-SE", "CCA-SE", "Abiotic-SE", "Coral-SE", "NatAlgae-SE"); full.SE.summary

# sample size 
full.n.summary<-aggregate(cbind(Inv_Algae, Ed, Ks, As, Gs, CCA, Abiotic, Coral, NatAlgae)~Reef+Treatment+Time, fig.df, length); colnames(full.n.summary) <- c("Reef", "Treatment", "Time", "InvAlgae-n", "ED-n", "KS-n", "AS-n", "GS-n", "CCA-n", "Abiotic-n", "Coral-n", "NatAlgae-n"); full.n.summary


#compiled summary mean, SE, n
full.data.summary<-data.frame(full.mean.summary[c(1:12,0)], full.SE.summary[c(0,4:12)], full.n.summary[c(0,5)]); colnames(full.data.summary) <- c("Reef", "Treatment", "Time", "InvAlgae-mean", "ES-mean", "KS-mean", "AS-mean", "GS-mean", "CCA-mean", "Abiotic-mean", "Coral-mean", "NatAlgae-mean", "InvAlgae-SE", "ED-SE", "KS-SE", "AS-SE", "GS-SE", "CCA-SE", "Abiotic-SE", "Coral-SE", "NatAlgae-SE", "N") ; full.data.summary


#export data summary
#write.csv(full.data.summary, "output/datasummary_updated.csv")
```

Create an individual dataframes of means and SE for figure generation

```
#####################################
## General formatting to apply to all figures
par(mfrow = c(1,1), mar=c(5,4,1,1))

pd <- position_dodge(0.1) #offset for error bars
 # theme position for legend in (x,y)
formatting<-(
  theme(panel.border = element_rect(fill=NA, color = "black", size = .1)) +
  theme(legend.key = element_blank()) +
  theme(legend.position = c(0.75,0.86)) +
  theme(text=element_text(size=10))+
  theme(axis.title.x = element_text(size=14))+
  theme(legend.text=element_text(size=10)) +
  theme(legend.title = element_blank())+
  theme(panel.background = element_rect(colour = "black", size=1))+ theme(aspect.ratio=1)+
  theme(axis.ticks.length=unit(-0.25, "cm"), 
        axis.text.y=element_text(margin=unit(c(0.5, 0.5, 0.5, 0.5), "cm")), 
        axis.text.x=element_text(margin=unit(c(0.5, 0.5, 0.5, 0.5), "cm"))))

# axis labels to replace "before and afters"
x.names<-c("Winter\n 2011","Summer\n 2012","Winter\n 2012", "Summer\n 2013", "Winter\n 2013")

############################
#Invasive Algae
Inv.mean<-aggregate(Inv_Algae~Treatment+Time, fig.df, mean)
Inv.SE<-aggregate(Inv_Algae~Treatment+Time, fig.df, std.error)
Inv.df<-data.frame(Inv.mean, Inv.SE[c(3,0)]); colnames(Inv.df) <- c("Treatment", "Time", "mean", "se")
Inv.df["Treat_Time"]<-paste(Inv.df$Treatment, Inv.df$Time)

Fig.InvAlg<-ggplot(data=Inv.df, aes(x=Time, y=mean, group=Treatment, fill=Treatment)) + geom_errorbar(aes(ymin=mean-se, ymax=mean+se),size=.5, width=0, position=pd) +
  geom_line(position=pd, size=.5) +
  coord_cartesian(ylim=c(0, 35)) +
  geom_point(aes(fill=Treatment), position=pd, size=4, pch=21) +
  scale_fill_manual(values=c("white","black"),
                    labels=c("Control", "Treatment")) +
  ylab("Invasive Algae (% cover)") +
  scale_x_discrete(labels=x.names)+
  xlab("Sampling Times") + theme_classic() +
  formatting
# ggsave(file="Fig.InvAlg.eps", dpi=300)

Fig.InvAlg
```

```
######################################
# CCA

CCA.mean<-aggregate(CCA~Treatment+Time, fig.df, mean)
CCA.SE<-aggregate(CCA~Treatment+Time, fig.df, std.error)
CCA.df<-data.frame(CCA.mean, CCA.SE[c(3,0)]); colnames(CCA.df) <- c("Treatment", "Time", "mean", "se")
CCA.df["Treat_Time"]<-paste(CCA.df$Treatment, CCA.df$Time)

# plot figure with hab-treat means +/- se using GGPLOT
Fig.CCA<-ggplot(data=CCA.df, aes(x=Time, y=mean, group=Treatment, fill=Treatment)) + geom_errorbar(aes(ymin=mean-se, ymax=mean+se),size=.5, width=0, position=pd) +
  geom_line(position=pd, size=.5) +
  coord_cartesian(ylim=c(0, 35)) +
  geom_point(aes(fill=Treatment), position=pd, size=4, pch=21) +
  scale_fill_manual(values=c("white","black"),
                    labels=c("Control", "Treatment")) +
  ylab("CCA (% cover)")+
  xlab("Sampling Times") + 
  scale_x_discrete(labels=x.names)+
  theme_classic() +
  formatting
# ggsave(file="Fig.CCA.eps", dpi=300)

Fig.CCA
```

```
######################################
# Abiotic

Abiot.mean<-aggregate(Abiotic~Treatment+Time, fig.df, mean)
Abiot.SE<-aggregate(Abiotic~Treatment+Time, fig.df, std.error)
Abiot.df<-data.frame(Abiot.mean, Abiot.SE[c(3,0)]); colnames(Abiot.df) <- c("Treatment", "Time", "mean", "se")
Abiot.df["Treat_Time"]<-paste(Abiot.df$Treatment, Abiot.df$Time)

# plot figure with hab-treat means +/- se using GGPLOT
Fig.Abiotic<-ggplot(data=Abiot.df, aes(x=Time, y=mean, group=Treatment, fill=Treatment)) + geom_errorbar(aes(ymin=mean-se, ymax=mean+se),size=.5, width=0, position=pd) +
  geom_line(position=pd, size=.5) +
  coord_cartesian(ylim=c(0, 70)) +
  geom_point(aes(fill=Treatment), position=pd, size=4, pch=21) +
  scale_fill_manual(values=c("white","black"),
                    labels=c("Control", "Treatment")) +
  ylab("Sand/Turf/Bare (% cover)") +
  xlab("Sampling Times") + 
  scale_x_discrete(labels=x.names)+
  theme_classic() +
  formatting
# ggsave(file="Fig.Abiotic.eps", dpi=300)

Fig.Abiotic
```

```
######################################
# Coral

Coral.mean<-aggregate(Coral~Treatment+Time, fig.df, mean)
Coral.SE<-aggregate(Coral~Treatment+Time, fig.df, std.error)
Coral.df<-data.frame(Coral.mean, Coral.SE[c(3,0)]); colnames(Coral.df) <- c("Treatment", "Time", "mean", "se")
Coral.df["Treat_Time"]<-paste(Coral.df$Treatment, Coral.df$Time)

# plot figure with hab-treat means +/- se using GGPLOT
Fig.Coral<-ggplot(data=Coral.df, aes(x=Time, y=mean, group=Treatment, fill=Treatment)) + geom_errorbar(aes(ymin=mean-se, ymax=mean+se),size=.5, width=0, position=pd) +
  geom_line(position=pd, size=.5) +
  coord_cartesian(ylim=c(0, 70)) +
  geom_point(aes(fill=Treatment), position=pd, size=4, pch=21) +
  scale_fill_manual(values=c("white","black"),
                    labels=c("Control", "Treatment")) +
  ylab("Coral (% cover)") +
  xlab("Sampling Times") + 
  scale_x_discrete(labels=x.names)+
  theme_classic() +
  formatting
# ggsave(file="Fig.Coral.eps", dpi=300)

Fig.Coral
```

```
######################################
# Native Algae

NatAlg.mean<-aggregate(NatAlgae~Treatment+Time, fig.df, mean)
NatAlg.SE<-aggregate(NatAlgae~Treatment+Time, fig.df, std.error)
NatAlg.df<-data.frame(NatAlg.mean, NatAlg.SE[c(3,0)]); colnames(NatAlg.df) <- c("Treatment", "Time", "mean", "se")
NatAlg.df["Treat_Time"]<-paste(NatAlg.df$Treatment, NatAlg.df$Time)

# plot figure with hab-treat means +/- se using GGPLOT
Fig.NatAlg<-ggplot(data=NatAlg.df, aes(x=Time, y=mean, group=Treatment, fill=Treatment)) + geom_errorbar(aes(ymin=mean-se, ymax=mean+se),size=.5, width=0, position=pd) +
  geom_line(position=pd, size=.5) +
  coord_cartesian(ylim=c(0, 35)) +
  geom_point(aes(fill=Treatment), position=pd, size=4, pch=21) +
  scale_fill_manual(values=c("white","black"),
                    labels=c("Control", "Treatment")) +
  ylab("Native Algae (% cover)") +
  xlab("Sampling Times") + 
  scale_x_discrete(labels=x.names)+
  theme_classic() +
  formatting
# ggsave(file="Fig.NatAlg.eps", dpi=300)

############
#export figures
#pdf(file="plots_5time points_InvAlg.pdf")
#grid.arrange(Fig.InvAlg)
#grid.arrange(Fig.CCA, Fig.NatAlg)
#grid.arrange(Fig.Coral, Fig.Abiotic)
#dev.off()
############

Fig.NatAlg
```

Generate a plot with All the invasive algae groups plotted individually at Control and Treatment Reefs through time.

```
#Ed dataframe
Ed.mean<-aggregate(Ed~Treatment+Time, fig.df, mean)
Ed.SE<-aggregate(Ed~Treatment+Time, fig.df, std.error)
Ed.df<-data.frame(Ed.mean, Ed.SE[c(3,0)]); colnames(Ed.df) <- c("Treatment", "Time", "mean", "se")
Ed.df["Treat_Time"]<-paste(Ed.df$Treatment, Ed.df$Time)

#Ks dataframe
Ks.mean<-aggregate(Ks~Treatment+Time, fig.df, mean)
Ks.SE<-aggregate(Ks~Treatment+Time, fig.df, std.error)
Ks.df<-data.frame(Ks.mean, Ks.SE[c(3,0)]); colnames(Ks.df) <- c("Treatment", "Time", "mean", "se")
Ks.df["Treat_Time"]<-paste(Ks.df$Treatment, Ks.df$Time)

#As dataframe
As.mean<-aggregate(As~Treatment+Time, fig.df, mean)
As.SE<-aggregate(As~Treatment+Time, fig.df, std.error)
As.df<-data.frame(As.mean, As.SE[c(3,0)]); colnames(As.df) <- c("Treatment", "Time", "mean", "se")
As.df["Treat_Time"]<-paste(As.df$Treatment, As.df$Time)

#Gs dataframe
Gs.mean<-aggregate(Gs~Treatment+Time, fig.df, mean)
Gs.SE<-aggregate(Gs~Treatment+Time, fig.df, std.error)
Gs.df<-data.frame(Gs.mean, Gs.SE[c(3,0)]); colnames(Gs.df) <- c("Treatment", "Time", "mean", "se")
Gs.df["Treat_Time"]<-paste(Gs.df$Treatment, Gs.df$Time)


######## dataframes ##################
# separating Control and Treatment dataframes
Ed.Cn<-Ed.df[(Ed.df$Treatment=="Control"),]
Ed.Tr<-Ed.df[(Ed.df$Treatment=="Treatment"),]

Ks.Cn<-Ks.df[(Ks.df$Treatment=="Control"),]
Ks.Tr<-Ks.df[(Ks.df$Treatment=="Treatment"),]

As.Cn<-As.df[(As.df$Treatment=="Control"),]
As.Tr<-As.df[(As.df$Treatment=="Treatment"),]

Gs.Cn<-Gs.df[(Gs.df$Treatment=="Control"),]
Gs.Tr<-Gs.df[(Gs.df$Treatment=="Treatment"),]


############## properties for plotting figure ############## 
Sample.Times=c(1,2,3,4,5)
Sample.Times2=c(1.02, 2.02, 3.02, 4.02, 5.02) # provides an offset from orignal x
Sample.Times3=c(0.95, 1.95, 2.95, 3.95, 4.95) # provides an offset from orignal x
Species=c(expression(italic("Eucheuma")~clade~E),
          expression(italic("Kappaphycus")~clade~B),
          expression(italic("Acanthophora spicifera")),
          expression(italic("Gracilaria salicornia")))
colors<-c("coral", "yellow3", "cadetblue3", "chartreuse4")


#######################################################
#### Control Reefs with all Invasive Algae species ####
#######################################################

par(mfrow = c(1,2), mar=c(5,4,1,1))

plot(Sample.Times, Ed.Cn$mean, ylab="Macroalgae (% cover)", xaxt="n", type="o", ylim=c(0,18), pch=19, xlab="Sampling Times", cex=1.5, lwd=2, col=colors[1], main="Control Reefs")
axis(side=1, at=Sample.Times, labels=x.names, cex.axis=0.8)
arrows(Sample.Times, Ed.Cn$mean-Ed.Cn$se, Sample.Times, Ed.Cn$mean+Ed.Cn$se, length=0, lwd=2, angle=90, code=3, col=colors[1])
with(Ks.Cn, lines(Sample.Times, mean, xaxt="n", type="o", pch=19, cex=1.5, lwd=2, col=colors[2]))
with(Ks.Cn, arrows(Sample.Times, mean-se, Sample.Times, mean+se, length=0, lwd=2, angle=90, code=3, col=colors[2]))
with(As.Cn, lines(x=Sample.Times2, y=mean, xaxt="n", type="o", pch=19, cex=1.5, lwd=2, col=colors[3]))
with(As.Cn, arrows(Sample.Times2, mean-se, Sample.Times2, mean+se, length=0, lwd=2, angle=90, code=3,col=colors[3]))
with(Gs.Cn, lines(Sample.Times3, mean, xaxt="n", type="o", pch=19, cex=1.5, lwd=2, col=colors[4]))
with(Gs.Cn, arrows(Sample.Times3, mean-se, Sample.Times3, mean+se, length=0, lwd=2, angle=90, code=3, col=colors[4]))


#########################################################
#### Treatment Reefs with all Invasive Algae species ####
#########################################################

plot(Sample.Times, Ed.Tr$mean, ylab="Macroalgae (% cover)",xlab="Sampling Times", xaxt="n", type="o", ylim=c(0,18), pch=19, cex=1.5, lwd=2, col=colors[1], main="Treatment Reefs")
axis(side=1, at=Sample.Times, labels=x.names, cex.axis=0.8)
arrows(Sample.Times, Ed.Tr$mean-Ed.Tr$se, Sample.Times, Ed.Tr$mean+Ed.Tr$se, length=0, lwd=2, angle=90, code=3, col=colors[1])
with(Ks.Tr, lines(Sample.Times2, mean, xaxt="n", type="o", pch=19, cex=1.5, lwd=2, col=colors[2]))
with(Ks.Tr, arrows(Sample.Times2, mean-se, Sample.Times2, mean+se, length=0, lwd=2, angle=90, code=3, col=colors[2]))
with(As.Tr, lines(Sample.Times2, mean, xaxt="n", type="o", pch=19, cex=1.5, lwd=2, col=colors[3]))
with(As.Tr, arrows(Sample.Times2, mean-se, Sample.Times2, mean+se, length=0, lwd=2, angle=90, code=3, col=colors[3]))
with(Gs.Tr, lines(Sample.Times3, mean, xaxt="n", type="o", pch=19, cex=1.5, lwd=2, col=colors[4]))
with(Gs.Tr, arrows(Sample.Times3, mean-se, Sample.Times3, mean+se, length=0, lwd=2, angle=90, code=3, col=colors[4]))
legend("topleft", inset=c(0.02, 0.01), legend=Species, col=colors, pch=19, pt.cex=1.5, cex=0.7, bty="n", x.intersp=0.9, y.intersp=1.3)
```

Generate a composition plot figure for Before and After for each reef and each treatment

```
# make dataframe with only rows and columns needed to make figure

##########  means by Reef ID and Time dataframe ########## 
comp.fig.df<-full.mean.summary[, c(1:4,9:12)] # just columns needing to graph
comp.fig.df<-comp.fig.df[(comp.fig.df$Time=="Before" | comp.fig.df$Time=="After4"),] # only show time points "Before" and Final "After4"

colnames(comp.fig.df)<-c("Reef", "Treatment", "Time", "Invasive Algae","CCA", "Sand/Bare/Turf", "Coral", "Native Algae")

# reorder columns
comp.fig.df<-comp.fig.df[, c(1:4, 8,5,7,6)]

# convert wide to long format data 
library("reshape2")
comp.reef.stack.fig<-melt(comp.fig.df, id.vars=1:3)
colnames(comp.reef.stack.fig)<-c("Reef", "Treatment", "Time", "member", "cover")

#colors
stack.palette<-c("darkgreen", "darkseagreen3", "lightcoral", "lightblue", "ivory")

# grouping factor for Time
comp.reef.stack.fig$Time<-factor(comp.reef.stack.fig$Time, levels=c("Before", "After4"))

#### Figxx 
# Fig.stack.reef.trt<-ggplot(comp.reef.stack.fig, aes(x = interaction(Reef, Treatment, lex.order=FALSE), y=cover, fill = member)) + 
#  geom_bar(position = "fill",stat = "identity", colour="black") + 
#  scale_y_continuous(labels = percent_format()) +
#  scale_fill_manual(values=stack.palette, labels=c("Invasive macroalgae", "Native macroalgae", "CCA", "Coral", #"SBT"),  guide = guide_legend(title = "Community members")) + 
#  scale_x_discrete(labels=c("Reef 16\nControl", "Reef 28\nControl", "Reef 26\nTreatment", "Reef 27\nTreatment", #"Reef 16\nControl", "Reef 28\nControl", "Reef 26\nTreatment", "Reef 27\nTreatment")) +
#  ggtitle("Benthic cover over time") +
#  theme(axis.text.x=element_text(size=6))+
#  xlab(expression(bold("Reef and Treatment"))) +
#  ylab(expression(bold(paste("Benthic cover %")))) + facet_grid(.~Time)

# Fig.stack.reef.trt
# ggsave(file="figures/Fig.stack.reef.trt.pdf")

########## ########## ########## ########## ########## ########## 
########## means  by Treatment and Time dataframe ##############
comp.fig.trt<-aggregate(cbind(Inv_Algae, CCA, Abiotic, Coral, NatAlgae)~Treatment+Time, fig.df, mean); colnames(comp.fig.trt) <- c("Treatment", "Time", "Invasive Algae","CCA", "Sand/Bare/Turf", "Coral", "Native Algae")
 
# remove times not wanted for figure
comp.fig.trt<-comp.fig.trt[(comp.fig.trt$Time=="Before" | comp.fig.trt$Time=="After4"),] # only show time points "Before" and Final "After4"

# reorder columns
comp.fig.trt<-comp.fig.trt[, c(1:3,7,4,6,5)]

# convert wide to long format data 
comp.fig.trt<-melt(comp.fig.trt, id.vars=1:2)
colnames(comp.fig.trt)<-c("Treatment", "Time", "member", "cover")

# grouping factor for Time
comp.fig.trt$Time<-factor(comp.fig.trt$Time, levels=c("Before", "After4"))

#### Fig 6
Fig.stack.trt<-ggplot(comp.fig.trt, aes(x =Treatment, y=cover, fill = member)) + 
  geom_bar(position = "fill", stat = "identity", colour="black") + 
  scale_y_continuous(labels = percent_format()) +
  scale_fill_manual(values=stack.palette, labels=c("Invasive macroalgae", "Native macroalgae", "CCA", "Coral", "SBT"),  guide = guide_legend(title = "Community members")) + 
  scale_x_discrete(labels=c("Control", "Algae removal\n + urchins")) +
  ggtitle("Benthic cover over time") +
  theme(axis.text.x=element_text(size=10))+
  xlab(expression(bold("Treatment"))) +
  ylab(expression(bold(paste("Benthic cover %")))) + facet_grid(.~Time)

Fig.stack.trt
```

```
# ggsave(file="figures/Fig.stack.trt.pdf")
```

## Statistical analysis

### Assumptions of ANOVA

First, check dataframe **ALLdata**. Make a new datarame named **df** and use this for diangostic plots, and run a for loop to insect data and model residuals. *(code below)*

```
################################################
################################################
# ALLdata = original unmodified proportion data
# use ALLdata dataframe for transformations and ANOVA assumptions
################################################
################################################

# reorganize columns in "ALLdata" and make "df" for subsequent use

df<-ALLdata[, c(1:7,18,8,10,11,15)]

################################################
# for loop for running model, inspecting residuals and diagnostic plots
for(i in 8:12){
  Y<-df[,i]
  full<-lmer(Y~Treatment*Time+(1|Reef/Habitat)+(1|Transect), data=df, na.action=na.exclude)
  R <- resid(full) #save glm residuals
  op<-par(mfrow = c(2,2), mar=c(5,4,1,2), pty="sq")
  plot(full, add.smooth = FALSE, which=1)
  QQ <- qqnorm(R, main = colnames(df)[i]) 
  QQline <- qqline(R)
  hist(R, xlab="Residuals", main = colnames(df)[i])
  plot(df$Treatment, R, xlab="Treatment", ylab="Residuals")
  plot(df$Time, R, xlab="Time", ylab="Residuals")
}
```

Most data has non-normal distribution, but *Coral cover* is normally distributed. ALl other metrics needs either an alternative model or a data transformation. To keep the nested structure of the data, transformations may be best option. Since data is proportion and distributed 0-1, an arc(sin) or square root transformation may improve.  
- Test *arc-sin squareroot* transformations *(code below)*

- **asin(sqrt(Y))** is good for some metrics *(Invasive algae (pooled), Abiotic)*
- The **square root (Y)** helps other data *(CCA, Native Algae)*
- Others are normally distributed *(Coral cover)*

### Model Selection

Testing for season effects, should it be in the model?

Results below show season has no effect and does not need to be included in the model.

```
#names(trans.df)
for(i in 8:12){
  var=trans.df[,i]
  mod<-lm(var~Season, data=trans.df)
#### compare models
  print(anova(mod))
}
```

```
## Analysis of Variance Table
## 
## Response: var
##            Df  Sum Sq  Mean Sq F value Pr(>F)
## Season      1  0.0209 0.020906  0.3451 0.5574
## Residuals 252 15.2645 0.060573               
## Analysis of Variance Table
## 
## Response: var
##            Df Sum Sq  Mean Sq F value Pr(>F)
## Season      1 0.0224 0.022428  0.6875 0.4078
## Residuals 252 8.2206 0.032622               
## Analysis of Variance Table
## 
## Response: var
##            Df Sum Sq  Mean Sq F value Pr(>F)
## Season      1  0.004 0.004022  0.0409   0.84
## Residuals 252 24.811 0.098456               
## Analysis of Variance Table
## 
## Response: var
##            Df  Sum Sq  Mean Sq F value Pr(>F)
## Season      1  0.0089 0.008871  0.0737 0.7863
## Residuals 252 30.3332 0.120370               
## Analysis of Variance Table
## 
## Response: var
##            Df Sum Sq   Mean Sq F value Pr(>F)
## Season      1 0.0022 0.0022254  0.1402 0.7084
## Residuals 252 4.0004 0.0158746
```

### Linear models

- remember to use the “trans.df” dataframe here, as this is the dataframe with transformed data.

#### Invasive Algae

```
####################
# Invasive Algae
####################
# dataframe for models is "trans.df"

full<-lmer(Inv.trans~Treatment*Time+(1|Reef/Habitat)+(1|Transect), data=trans.df)
# summary(full)
anova(full, type=2) # THIS is the stats table
```

```
## Analysis of Variance Table of type II  with  Satterthwaite 
## approximation for degrees of freedom
##                 Sum Sq Mean Sq NumDF  DenDF F.value    Pr(>F)    
## Treatment      0.03090 0.03090     1   9.44   3.377   0.09773 .  
## Time           1.47799 0.36950     4 195.04  40.389 < 2.2e-16 ***
## Treatment:Time 0.62948 0.15737     4 195.04  17.202 4.213e-12 ***
## ---
## Signif. codes:  0 '***' 0.001 '**' 0.01 '*' 0.05 '.' 0.1 ' ' 1
```

```
# difflsmeans(full, test.effs="Treatment:Time", adjust=Tukey)

detach("package:lmerTest", unload=TRUE)
posthoc<-lsmeans(full, pairwise~Treatment|Time)
cld(posthoc, Letters=letters)
```

```
## Time = Before:
##  Treatment     lsmean         SE    df     lower.CL  upper.CL .group
##  Treatment 0.42372061 0.06822913 10.37  0.273101548 0.5743397  a    
##  Control   0.44722332 0.07601657 10.74  0.279413120 0.6150335  a    
## 
## Time = After1:
##  Treatment     lsmean         SE    df     lower.CL  upper.CL .group
##  Treatment 0.32752125 0.06822913 10.37  0.176902190 0.4781403  a    
##  Control   0.43512659 0.07601657 10.74  0.267316390 0.6029368  a    
## 
## Time = After2:
##  Treatment     lsmean         SE    df     lower.CL  upper.CL .group
##  Treatment 0.23771653 0.06822913 10.37  0.087097474 0.3883356  a    
##  Control   0.49586948 0.07601657 10.74  0.328059281 0.6636797   b   
## 
## Time = After3:
##  Treatment     lsmean         SE    df     lower.CL  upper.CL .group
##  Treatment 0.15192888 0.06822913 10.37  0.001309817 0.3025479  a    
##  Control   0.40082138 0.07601657 10.74  0.233011174 0.5686316   b   
## 
## Time = After4:
##  Treatment     lsmean         SE    df     lower.CL  upper.CL .group
##  Treatment 0.09232811 0.06835166 10.44 -0.058561436 0.2432177  a    
##  Control   0.36816346 0.07601657 10.74  0.200353257 0.5359737   b   
## 
## Degrees-of-freedom method: satterthwaite 
## Confidence level used: 0.95 
## significance level used: alpha = 0.05
```

#### Coral

```
####################
# Coral 
library('lmerTest')
####################
# random intercepts and random Treatment slope 
full<-lmer(Coral.trans~Treatment*Time+(1|Reef/Habitat)+(1|Transect), data=trans.df, na.action=na.exclude)

# summary(full)
anova(full, type=2)
```

```
## Analysis of Variance Table of type II  with  Satterthwaite 
## approximation for degrees of freedom
##                  Sum Sq  Mean Sq NumDF   DenDF F.value    Pr(>F)    
## Treatment      0.000073 0.000073     1   9.048   0.056  0.817788    
## Time           0.181239 0.045310     4 195.073  34.783 < 2.2e-16 ***
## Treatment:Time 0.020148 0.005037     4 195.070   3.867  0.004785 ** 
## ---
## Signif. codes:  0 '***' 0.001 '**' 0.01 '*' 0.05 '.' 0.1 ' ' 1
```

```
# difflsmeans(full, test.effs="Treatment:Time", adjust=Tukey)

detach("package:lmerTest", unload=TRUE)
posthoc<-lsmeans(full, pairwise~Treatment|Time)
cld(posthoc, Letters=letters)
```

```
## Time = Before:
##  Treatment    lsmean        SE   df    lower.CL  upper.CL .group
##  Treatment 0.3470514 0.1512674 9.07 0.005365835 0.6887369  a    
##  Control   0.4297208 0.1658077 9.09 0.055191245 0.8042503  a    
## 
## Time = After1:
##  Treatment    lsmean        SE   df    lower.CL  upper.CL .group
##  Treatment 0.3796792 0.1512674 9.07 0.037993701 0.7213648  a    
##  Control   0.4343504 0.1658077 9.09 0.059820875 0.8088799  a    
## 
## Time = After2:
##  Treatment    lsmean        SE   df    lower.CL  upper.CL .group
##  Treatment 0.4046645 0.1512674 9.07 0.062979004 0.7463501  a    
##  Control   0.4350118 0.1658077 9.09 0.060482250 0.8095413  a    
## 
## Time = After3:
##  Treatment    lsmean        SE   df    lower.CL  upper.CL .group
##  Treatment 0.3826187 0.1512674 9.07 0.040933148 0.7243042  a    
##  Control   0.4406335 0.1658077 9.09 0.066103944 0.8151630  a    
## 
## Time = After4:
##  Treatment    lsmean        SE   df    lower.CL  upper.CL .group
##  Treatment 0.4478915 0.1512753 9.07 0.106188133 0.7895949  a    
##  Control   0.4879218 0.1658077 9.09 0.113392303 0.8624514  a    
## 
## Degrees-of-freedom method: satterthwaite 
## Confidence level used: 0.95 
## significance level used: alpha = 0.05
```

**CCA**

```
####################
# CCA
library('lmerTest')
####################
full<-lmer(CCA.trans~Treatment*Time+(1|Reef/Habitat)+(1|Transect), data=trans.df, na.action=na.exclude)

# summary(full)
anova(full, type=2)
```

```
## Analysis of Variance Table of type II  with  Satterthwaite 
## approximation for degrees of freedom
##                 Sum Sq  Mean Sq NumDF   DenDF F.value    Pr(>F)    
## Treatment      0.00045 0.000446     1   9.234  0.0448   0.83700    
## Time           0.36611 0.091527     4 195.118  9.1937 7.958e-07 ***
## Treatment:Time 0.10378 0.025946     4 195.113  2.6062   0.03707 *  
## ---
## Signif. codes:  0 '***' 0.001 '**' 0.01 '*' 0.05 '.' 0.1 ' ' 1
```

```
# difflsmeans(full, test.effs="Treatment:Time", adjust=Tukey)

detach("package:lmerTest", unload=TRUE)
posthoc<-lsmeans(full, pairwise~Treatment|Time)
cld(posthoc, Letters=letters)
```

```
## Time = Before:
##  Treatment    lsmean         SE    df   lower.CL  upper.CL .group
##  Treatment 0.1438659 0.05514784 11.02 0.02293476 0.2647971  a    
##  Control   0.1689543 0.06122437 11.34 0.03469820 0.3032104  a    
## 
## Time = After1:
##  Treatment    lsmean         SE    df   lower.CL  upper.CL .group
##  Treatment 0.2128153 0.05514784 11.02 0.09188418 0.3337465  a    
##  Control   0.2180620 0.06122437 11.34 0.08380589 0.3523181  a    
## 
## Time = After2:
##  Treatment    lsmean         SE    df   lower.CL  upper.CL .group
##  Control   0.1863114 0.06122437 11.34 0.05205525 0.3205675  a    
##  Treatment 0.2786011 0.05514784 11.02 0.15766995 0.3995323  a    
## 
## Time = After3:
##  Treatment    lsmean         SE    df   lower.CL  upper.CL .group
##  Control   0.2566477 0.06122437 11.34 0.12239158 0.3909038  a    
##  Treatment 0.2585216 0.05514784 11.02 0.13759048 0.3794528  a    
## 
## Time = After4:
##  Treatment    lsmean         SE    df   lower.CL  upper.CL .group
##  Control   0.2485502 0.06122437 11.34 0.11429409 0.3828063  a    
##  Treatment 0.2678387 0.05531026 11.15 0.14655138 0.3891260  a    
## 
## Degrees-of-freedom method: satterthwaite 
## Confidence level used: 0.95 
## significance level used: alpha = 0.05
```

#### Native Algae

```
####################
# Native Algae
library('lmerTest')
####################
full<-lmer(NatAlg.trans~Treatment*Time+(1|Reef/Habitat)+(1|Transect), data=trans.df, na.action=na.exclude)

# summary(full)
anova(full, type=2)
```

```
## Analysis of Variance Table of type II  with  Satterthwaite 
## approximation for degrees of freedom
##                  Sum Sq   Mean Sq NumDF   DenDF F.value    Pr(>F)    
## Treatment      0.000050 0.0000495     1   9.134  0.0146    0.9063    
## Time           0.119538 0.0298846     4 195.244  8.8406 1.399e-06 ***
## Treatment:Time 0.026075 0.0065187     4 195.233  1.9284    0.1072    
## ---
## Signif. codes:  0 '***' 0.001 '**' 0.01 '*' 0.05 '.' 0.1 ' ' 1
```

```
# difflsmeans(full, test.effs="Treatment:Time", adjust=Tukey)

detach("package:lmerTest", unload=TRUE)
posthoc<-lsmeans(full, pairwise~Treatment|Time)
cld(posthoc, Letters=letters)
```

```
## Time = Before:
##  Treatment     lsmean         SE    df     lower.CL  upper.CL .group
##  Control   0.16420960 0.05050659 10.07  0.051777037 0.2766422  a    
##  Treatment 0.16762638 0.04598801  9.97  0.065252608 0.2700001  a    
## 
## Time = After1:
##  Treatment     lsmean         SE    df     lower.CL  upper.CL .group
##  Control   0.14440302 0.05050659 10.07  0.031970454 0.2568356  a    
##  Treatment 0.16363483 0.04598801  9.97  0.061261062 0.2660086  a    
## 
## Time = After2:
##  Treatment     lsmean         SE    df     lower.CL  upper.CL .group
##  Treatment 0.14672492 0.04598801  9.97  0.044351156 0.2490987  a    
##  Control   0.14864030 0.05050659 10.07  0.036207738 0.2610729  a    
## 
## Time = After3:
##  Treatment     lsmean         SE    df     lower.CL  upper.CL .group
##  Treatment 0.10705797 0.04598801  9.97  0.004684205 0.2094317  a    
##  Control   0.13012944 0.05050659 10.07  0.017696875 0.2425620  a    
## 
## Time = After4:
##  Treatment     lsmean         SE    df     lower.CL  upper.CL .group
##  Treatment 0.09102489 0.04605151 10.03 -0.011490233 0.1935400  a    
##  Control   0.12960749 0.05050659 10.07  0.017174926 0.2420400  a    
## 
## Degrees-of-freedom method: satterthwaite 
## Confidence level used: 0.95 
## significance level used: alpha = 0.05
```

#### Abiotic

```
####################
# Abiotic
library('lmerTest')
####################
full<-lmer(Abio.trans~Treatment*Time+(1|Reef/Habitat)+(1|Transect), data=trans.df, na.action=na.exclude)

# summary(full)
anova(full, type=2)
```

```
## Analysis of Variance Table of type II  with  Satterthwaite 
## approximation for degrees of freedom
##                  Sum Sq   Mean Sq NumDF   DenDF F.value Pr(>F)
## Treatment      0.006966 0.0069658     1   8.992 0.51976 0.4893
## Time           0.072061 0.0180152     4 195.187 1.34422 0.2550
## Treatment:Time 0.047886 0.0119715     4 195.175 0.89326 0.4690
```

```
# difflsmeans(full, test.effs="Treatment:Time", adjust=Tukey)

detach("package:lmerTest", unload=TRUE)
posthoc<-lsmeans(full, pairwise~Treatment|Time)
cld(posthoc, Letters=letters)
```

```
## Time = Before:
##  Treatment    lsmean        SE   df  lower.CL  upper.CL .group
##  Control   0.5220554 0.1476818 9.40 0.1901358 0.8539749  a    
##  Treatment 0.6207030 0.1346729 9.36 0.3180215 0.9233846  a    
## 
## Time = After1:
##  Treatment    lsmean        SE   df  lower.CL  upper.CL .group
##  Control   0.4835475 0.1476818 9.40 0.1516279 0.8154670  a    
##  Treatment 0.6177565 0.1346729 9.36 0.3150750 0.9204381  a    
## 
## Time = After2:
##  Treatment    lsmean        SE   df  lower.CL  upper.CL .group
##  Control   0.4465420 0.1476818 9.40 0.1146224 0.7784615  a    
##  Treatment 0.5940945 0.1346729 9.36 0.2914129 0.8967760  a    
## 
## Time = After3:
##  Treatment    lsmean        SE   df  lower.CL  upper.CL .group
##  Control   0.4816033 0.1476818 9.40 0.1496838 0.8135228  a    
##  Treatment 0.6304689 0.1346729 9.36 0.3277874 0.9331505  a    
## 
## Time = After4:
##  Treatment    lsmean        SE   df  lower.CL  upper.CL .group
##  Control   0.4450180 0.1476818 9.40 0.1130985 0.7769375  a    
##  Treatment 0.6293096 0.1347583 9.39 0.3264362 0.9321830  a    
## 
## Degrees-of-freedom method: satterthwaite 
## Confidence level used: 0.95 
## significance level used: alpha = 0.05
```
